# Global research trends on the links between gut microbiota and radiotherapy: a bibliometric analysis (2004-2023)

**DOI:** 10.3389/fcimb.2024.1414196

**Published:** 2024-09-04

**Authors:** Shuyuan Wang, Zhen Yuan, Xiaohui Gao, Jiaxing Wu, Yifan Ren, Xiufeng Yu, Jianxiong Li, Wei Wei

**Affiliations:** ^1^ School of Medicine, Nankai University, Tianjin, China; ^2^ Department of Radiotherapy, Chinese PLA General Hospital, Beijing, China; ^3^ Department of Oncology, The Nuclear Industry 416 Hospital, Chengdu, China; ^4^ Tuberculosis Hospital of Shaanxi Province, Xi’an, China

**Keywords:** gut microbiota, cancer, radiotherapy, radiosensitivity, gut-organ axis

## Abstract

**Background:**

There is a crosstalk between gut microbiota and radiotherapy. The aim of this study is to use bibliometric analysis to explore the research status and development trends of research on gut microbiota and radiotherapy.

**Methods:**

A literature search regarding publications on gut microbiota and radiotherapy from 2004 to 2023 was retrieved. CiteSpace and VOSviewer were used to conduct the bibliometric analysis. The growth rate of publications, leading countries and institutions, preferred journals, top authors and co-cited authors, top co-cited references, keywords and citation were analyzed in this study.

**Results:**

A total of 2821 papers were extracted. The number of papers has increased rapidly over the past decade, especially after 2017. The USA and China had the most publications and made great contributions to this field. The Chinese Academy of Sciences stood out as the institution with the highest number of publications, followed by the Chinese Academy of Medical Sciences & Peking Union Medical College. The most influential authors were Fan Saijun and Li Yuan. *PLoS One* had the most publications and the most total citations. Highly cited papers and high-frequency keywords illustrated the current status and trends. Furthermore, analysis of keyword with burst revealed that *immunotherapy*, *acid*, *intestinal barrier, therapy*, *immunotherapy*, *fecal microbiota transplantation*, etc, are at the forefront of research in this area.

**Conclusion:**

This study provides an overview of research on gut microbiota and radiotherapy, highlighting influential contributors, impactful publications, and emerging trends. Our finding suggests avenues for further exploration to improve clinical outcomes.

## 1 Introduction

In recent decades, gut microbiota, a key regulator of host metabolism and immunity, has gained attention for its role in modulating radiation effects. This complex microbial community exhibits a bidirectional interaction with radiotherapy, influencing both the treatment’s efficacy and associated radiation injury (RI) (Dong et al., 2021; Li et al., 2022; Wang et al., 2023a).

Emerging research has demonstrated that gut microbiota may enhance the radiosensitivity (Crawford and Gordon, 2005; Paulos et al., 2007; Cui et al., 2016). A preclinical study has suggested a correlation between the gut microbiota and reduced efficacy of radiotherapy in patients treated with antibiotics (Nenclares et al., 2020). Nevertheless, with limited data available, the gut microbiota has also been implicated in the modulation of resistance to radiotherapy (Yang et al., 2019; Yang et al., 2022). For example, turmeric powder has been found to protect the gut microbiota from imbalances caused by radiotherapy, which partly explains the pharmacodynamic mechanism of turmeric powder in radio sensitization, and indirectly reflects the negative correlation between intestinal microbiota imbalance and therapeutic effect (Yang et al., 2019).

In addition, gut microbiota has also been shown to be involved in RI, spanning across various organs, including the skin (Ramadan et al., 2021; Trompette et al., 2022), brain (Liu et al., 2019; Song et al., 2022), lung (Chen et al., 2021), heart (Bartolomaeus et al., 2019; Chen et al., 2021), gastrointestinal tract (Reis Ferreira et al., 2019; Wang et al., 2023c), liver (Miousse et al., 2020), and hematopoietic system (Lucas et al., 2018; Guo et al., 2020; Lee et al., 2021). Molecular mechanisms such as DNA damage, oxidative stress, and inflammation underlie these radiation effects, with the gut microbiota playing a critical role in modulating these pathways (Russo et al., 2019; Nishiyama et al., 2021; Liu et al., 2022). However, emerging evidence suggests that interventions targeting gut microbiota, such as fecal microbiota transplantation, probiotics, and prebiotics, offer promising avenues for mitigating RI (Schuijt et al., 2016; Chen et al., 2021; Zeng et al., 2023). These approaches aim to restore gut microbiota diversity, strengthen the intestinal barrier, and modulate immune responses, thereby reducing the severity of radiation-induced damage.

Bibliometric analysis has emerged as a powerful method for examining research trends and the impact of scientific publications (Noda et al., 2023; Su et al., 2023; Wang et al., 2023b). This approach involves quantitatively assessing various aspects of scholarly work, such as publication output, citation patterns, and collaboration networks. However, there is currently no article on the quantitative analysis of interactions between gut microbiota and radiotherapy. Therefore, by employing bibliometric techniques, the aim of this study include: (1) summarizing the historical features of literature on gut microbiota and radiotherapy; (2) highlighting articles that have made significant contributions to the field; (3) recognizing the active topics of the research field; and (4) revealing emerging trends for future research.

## 2 Methods

### 2.1 Data sources and search strategies

The Web of Science Core Collection (WoSCC) database is widely regarded as the optimal choice for literature analysis due to its superior accuracy in labeling literature types (Thelwall, 2008; Merigó and Yang, 2017). We retrieved all relevant articles in WoSCC, within the timeframe from January 1, 2004 to November 29, 2023. The search strategy applied was tab search (TS) = “radiotherapy” and “gut microbiota” and their synonyms. The detailed search strategy is in **Supplementary Table 1**. The literature selection of this study is based on the following inclusion criteria: (1) The period of the literature search was from January 1, 2004 to November 28, 2023; (2) The language type was set to only English; (3) Document types were limited to “article” and “review”. During the process of reviewing the articles, any identified articles referenced were included as well. Two researchers (SW and XG) independently performed the search and data extraction. All searches were performed on the same day (November 28, 2023) to avoid the bias caused by database updates. All available information such as title, author, institution, country, publication year, and keywords from the raw data were collected. The WoSCC was used to download all records as plain text file exported.

### 2.2 Data analysis

GraphPad Prism v8.0.2 (GraphPad Software, Boston, USA) was used to analyze and plot annual paper publication trends and ratios at the national level. CiteSpace (version 6.1.6R, advanced edition) which was developed at Leiden University’s Centre for Science and Technology Studies, and VOSviewer (version 1.6.18), which was developed by Chaomei Chen at Drexel University, Philadelphia, United States, were used for data analysis and to visualize the scientific knowledge map. CiteSpace is widely used software for academic literature analysis, capable of generating temporal network maps that highlight key paths and evolutionary trends within the literature (Chen, 2006; Chen and Leydesdorff, 2014). VOSviewer v1.6.17, developed by Waltman et al. in 2009, is a free JAVA-based software used for analyzing extensive literature data and displaying it in map format (Van Eck and Waltman, 2010; Van Eck and Waltman, 2017). To visualize research achievements in a specific field by plotting a co-citation network of papers, professor Chen created the software CiteSpace (version 6.1.6R). This software is designed to explore new concepts and evaluate existing technologies within an experimental framework. It empowers users to gain a deeper understanding of knowledge domains, research frontiers, trends, and anticipate future advancements in research. All single images are provided as supplementary files (**Supplementary Data Sheets 1**, **2**).

Ethical approval was waived by the local ethical board because the data came from public databases, and no human or animal subjects were involved.

## 3 Results

### 3.1 An overview of the annual growth trend

**Figure 1:**
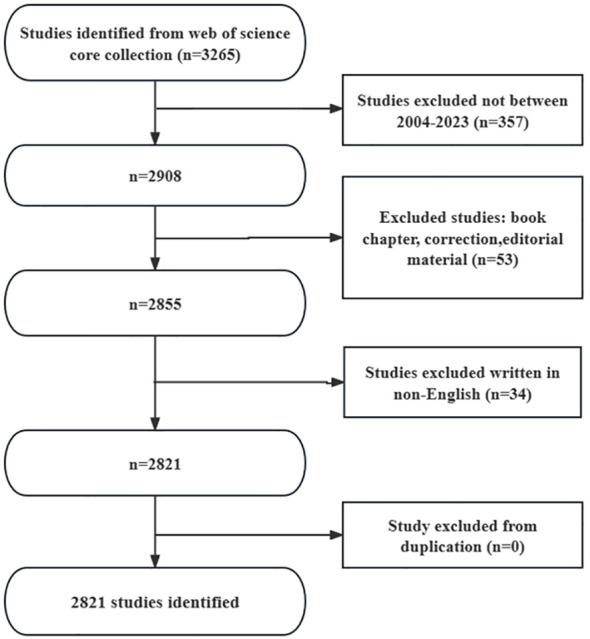
Flow diagram of the inclusion process. The detailed process of screening and enrollment.

After screening, our study identified a total of 2821 literature articles concerning on gut microbiota and radiotherapy in the WoSCC database (**Figure 1**), comprising 2410 articles (85.43%) and 415 reviews (14.57%). These articles spanned the period from January 1, 2004 to November 29, 2023, contributions from 114 countries and regions, 3,395 institutions, and 14,351 authors.

**Figure 2:**
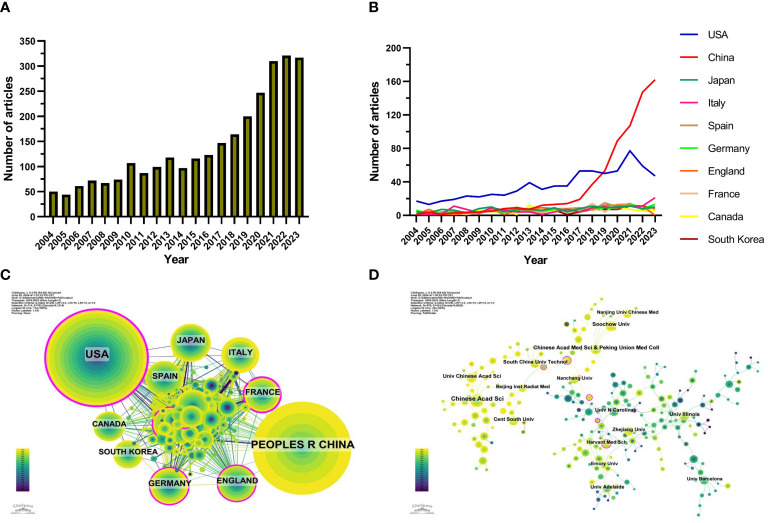
**(A)** Number of publications per year from 2004 to 2023 based on results following the strategy in **Figure 1** **(B)** Number of publications per country from 2004 to 2023. **(C)** A Cooperation map of countries obtained with Cite-Space. The size of the nodes’ concentric circles represents the publication volume of each country; the larger the circle, the greater the publication volume. The colors from purple to yellow correspond to the annual time periods of publications from 2004 to 2023. The outermost purple ring indicates the extent of the node’s connections with other nodes, with a wider purple ring signifying higher centrality. **(D)** The cooperation network map of institutions generated by CiteSpace. Each node represents an institution and the node size is proportional to the number of publications by that institution. The connecting line between nodes indicates the cooperation relationship, and the value of link strength is also displayed between lines. The colors from purple to yellow correspond to the annual time periods of publications from 2004 to 2023.

Starting from 2004, there has been a gradual increase in the annual number of publications (**Figure 2A**). This upward trend can be divided into three phases: a slow growth period from 2004 to 2010, with fewer than 100 papers published annually, suggesting limited attention to this field. The second phase from 2010 to 2017 saw a gradual increase in publications, suggesting the growing interest in this field. After 2017, a rapid increase in publications was observed, peaking in 2022, indicating widespread attention to this field after 2017.

### 3.2 Distribution and cooperation between countries/regions and institutions

Research on gut microbiota and radiotherapy has been conducted in 114 countries and regions. **Figure 2B** shows the trend in annual publication volume for the top 10 countries over the past two decades. The top five countries ranked by the quantity of articles in this field were the USA, China, Japan, Italy, and Spain (**Table 1**). Notably, the USA and China combined accounted for 50.30% of the total publications, substantially exceeding other countries.

**Table 1:** Top ten countries in research on gut microbiota and radiotherapy.

Rank	Country	Article counts	Centrality	Percentage (%)	Citation	Citation per publication
1	USA	721	0.47	25.56	30052	41.62
2	China	698	0.09	24.74	11270	16.12
3	Japan	145	0.10	5.14	5161	35.59
4	Italy	134	0.06	4.75	5859	43.72
5	Spain	131	0.06	4.64	4199	32.05
6	Germany	130	0.15	4.61	4272	32.86
7	England	122	0.12	4.32	4210	34.51
8	France	121	0.23	4.29	5545	45.83
9	Canada	106	0.04	3.76	2399	22.63
10	South Korea	102	0.00	3.62	2509	24.60

In the top ten publishing countries and regions, the USA led with 30,052 citations, ranking second in citations per publication (41.62). China, with the second-highest publication volume (698), had the second-highest citation volume (11,270) but the lowest citations per publication (16.12) among the top ten countries and regions, suggesting generally lower quality of publications.

The cooperation map of countries (**Figure 2C**) demonstrated a close collaboration between the highest-producing countries, the USA and China, with the USA also closely collaborating with France, Germany, and the United Kingdom, while China’s collaborations were more extensive with Japan, Canada, and South Korea. The centrality of 0.47 for the USA highlights its leading position in this field.

Of the 3,395 institutions publishing articles on gut microbiota and radiotherapy, the top ten institutions included seven from China, two from the USA, and one from Australia (**Table 2**; **Figure 2D**). The Chinese Academy of Sciences stood out as the institution with the highest number of publications (36 papers, 620 citations, averaging 17.22 citations per paper), followed by the Chinese Academy of Medical Sciences & Peking Union Medical College (22 papers, 314 citations, averaging 14.27 citations per paper), and Soochow University (21 papers, 175 citations, averaging 8.33 citations per paper). These findings indicated a preference for institutions to collaborate within their own countries, highlighting the need for enhanced international collaboration to break academic barriers.

**Table 2:** Top ten institutions in research on gut microbiota and radiotherapy.

Rank	Institution	Country	Number of studies	Total citations	Average citation
1	Chinese Acad Sci	China	36	620	17.22
2	Chinese Acad Med Sci & Peking Union Med Coll	China	22	314	14.27
2	Soochow Univ	China	21	175	8.33
4	Univ Chinese Acad Sci	China	20	188	9.40
5	Univ Illinois	USA	19	681	35.84
6	South China Univ Technol	China	18	300	16.67
7	Univ N Carolina	USA	18	1563	86.83
8	Univ Adelaide	Australia	18	886	49.22
9	Cent South Univ	China	18	125	6.94
10	Zhejiang Univ	China	16	431	26.94

### 3.3 Analysis of journals

**Figure 3:**
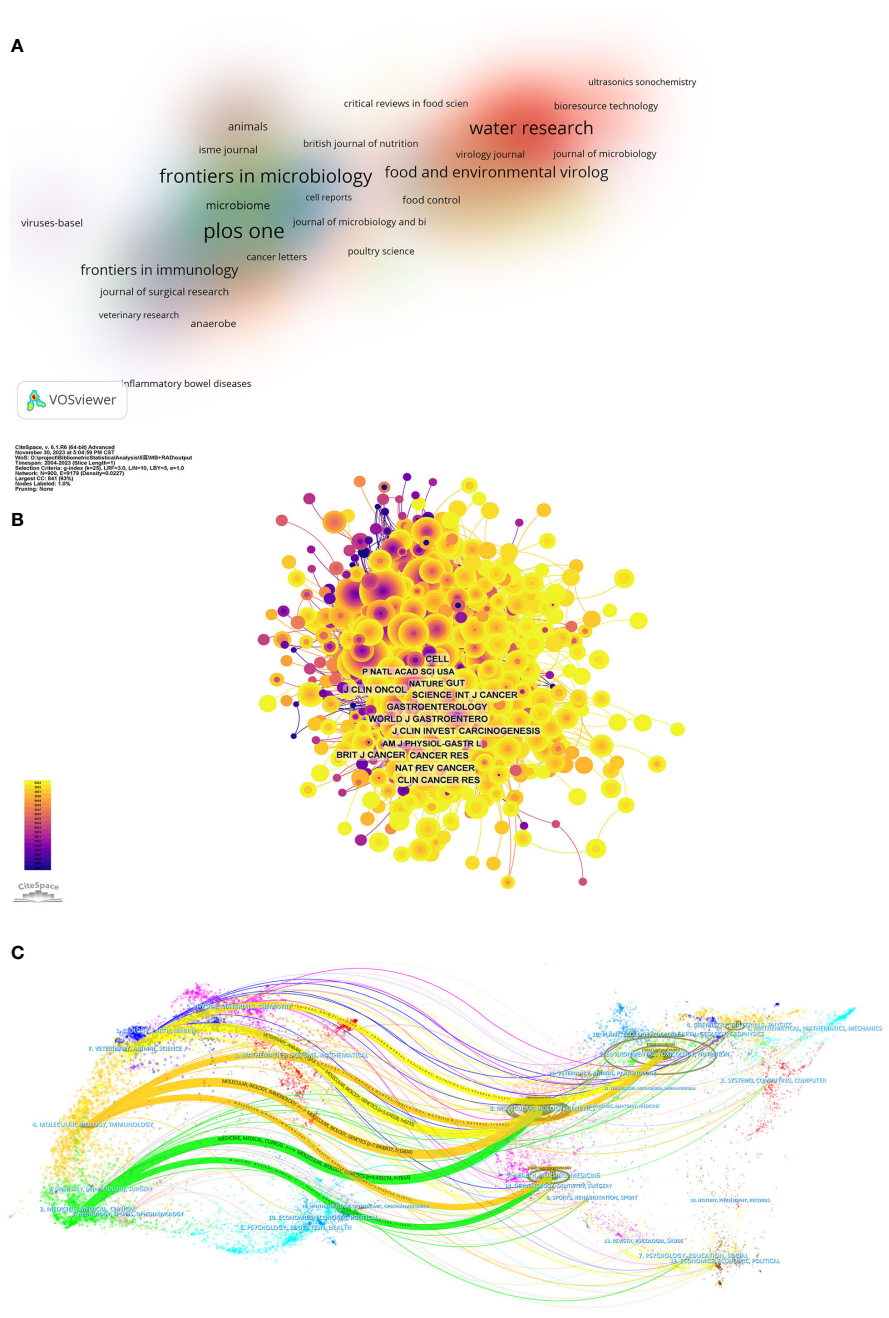
**(A)** Density visualization map of productive journals obtained with VOS Viewer. Colors represent clusters automatically calculated by VOSviewer. **(B)** Network map of co-cited journals obtained with Cite-Space, with the highest font size journal. The nodes represent co-cited journals. The lines between the nodes represent co-citation relationships. The larger the node area, the larger the number of co-citations. The colors from purple to yellow correspond to the annual time periods of publications from 2004 to 2023. **(C)** The dual-map overlay of journals obtained with Cite-Space. Each dot on the map represents a journal, with journals citing the references located on the left side and journals being cited located on the right side. The weight given to the color of a certain cluster is determined by the number of items belonging to that cluster in the neighborhood of the point, with each color indicating each cluster.

**Figure 3A** shows the density visualization map of productive journals, with the top ten productive journals listed in **Table 3**. PLoS One (80 publications, 2.13%) is the most productive journal in this field, followed by Front Microbiol (60 publications, 2.13%), Water Res (46 publications, 1.63%). Among the top ten productive journals, Water Res had the highest impact factor (IF) of 12.8, with all journals classified within Q1 or Q2 categories.

**Table 3:** Top ten productive journals in research on gut microbiota and radiotherapy.

Rank	Journal	Article counts	Percentage(867)	IF	Quartile in category
1	PLoS One	80	2.84	3.7	Q2
2	Front Microbiol	60	2.13	5.2	Q2
3	Water Res	56	1.99	12.8	Q1
4	Int J Mol Sci	46	1.63	5.6	Q1
5	Appl Environ Microbiol	40	1.42	4.4	Q2
6	Sci Total Environ	39	1.38	9.8	Q1
7	Food Environ Virol	34	1.21	3.4	Q2
8	Front Immunol	28	0.99	7.3	Q1
9	Int J Food Microbiol	28	0.99	5.4	Q1
10	Sci Rep	27	0.96	4.6	Q2

IF, impact factors.

The influence of a journal is determined by the frequency of its co-citations, indicating its significant impact on the scientific community. As shown in **Figure 3B** and **Table 4**, the journal most frequently co-cited is PLoS One (1234 times), followed by Appl Environ Microb (1128 times) and P Natl Acad Sci USA (1049 times). Among the top 10 most co-cited journals, Nature stands out with 1033 citations, possessing the highest IF (64.8). All these journals with high co-citation frequencies are ranked in either the Q1 or Q2 categories.

**Table 4:** Top ten co-cited journals in research on gut microbiota and radiotherapy.

Rank	Cited Journal	Co-Citation	IF(2020)	Quartile in category
1	PLoS One	1234	3.7	Q2
2	Appl Environ Microb	1128	4.4	Q2
3	P Natl Acad Sci USA	1049	11.1	Q1
4	Nature	1033	64.8	Q1
5	Science	971	56.9	Q1
6	Gut	792	24.5	Q1
7	Gastroenterology	748	29.4	Q1
8	Sci Rep-UK	701	4.6	Q2
9	Cell	605	64.5	Q1
10	Front Microbiol	573	5.2	Q2

The dual map overlay is an analytical method that shows domain-level citation concentration with their reference paths. As shown in **Figure 3C**, the colored path represents the citation relationship, with the citing journals on the left and the cited journals on the right. We identified seven primary citation paths. Studies published in the molecular/biology/immunology fields are mainly cited by research in molecular/biology/genetics, environmental/toxicology/nutrition, and health/nursing/medicine fields. studies from medicine/medical/clinical fields are predominantly cited by research in molecular/biology/genetics and health/nursing/medicine. Furthermore, studies published in the veterinary/animal/science field are mainly cited by research in molecular/biology/genetics and environmental/toxicology/nutrition fields.

### 3.4 Analysis of authors

**Figure 4:**
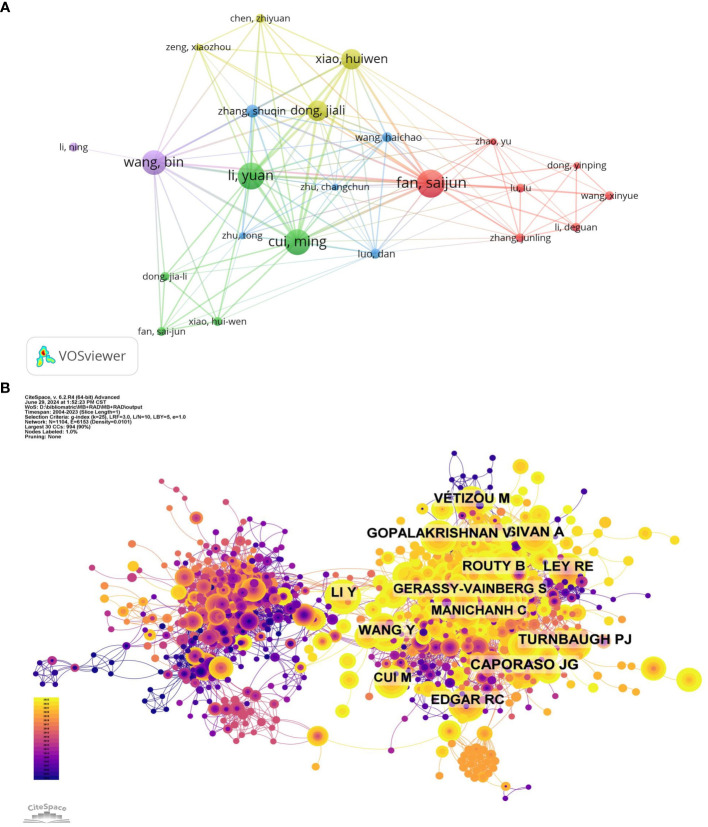
**(A)** Cooperation network of productive authors obtained with VOS Viewer. The nodes in the map represent authors, and lines between the nodes represent the collaborative relationships. The larger the number of publications, the larger the node area. Colors represent clusters automatically calculated by VOSviewer. **(B)** Network map of co-cited authors obtained with Cite-Space. The nodes represent co-cited authors. The lines between the nodes represent co-citation relationships. The larger the node area, the larger the number of co-citations. The colors from purple to yellow correspond to the annual time periods of publications from 2004 to 2023.

Among authors contributing to this field, the top ten published a total of 169 papers accounting for 5.99% of all publications. Fan Saijun has the highest number of research publications (21 publications), followed by Li Yuan (20 publications) and Cui Ming (19 publications) (**Table 5**; **Figure 4A**). Further analysis reveals that six of the top ten authors were from China, three from Japan, and one from the USA. Network visualization by CiteSpace showed these authors’ collaborative relationships (**Figure 4B**).

**Table 5:** Top ten productive authors and co-cited authors in research on gut microbiota and radiotherapy.

Rank	Author	Count	Location	Rank	Co-cited author	Citation
1	Fan Saijun	21	China	1	Caporaso JG	124
2	Li Yuan	20	China	2	Turnbaugh PJ	108
3	Cui Ming	19	China	3	Sivan A	105
4	Nomoto Koji	18	Japan	4	Routy B	102
5	Wang Bin	18	China	5	Wang Y	102
6	Asahara Takashi	17	Japan	6	Ley RE	100
7	Hauer-Jensen Martin	15	USA	7	Edgar RC	96
8	Dong Jiali	14	China	8	Gopalakrishnan V	95
9	Xiao Huiwen	14	China	9	Li Y	94
10	Tsuji Hirokazu	13	Japan	10	Vetizou M	91

### 3.5 Analysis of references

**Figure 5:**
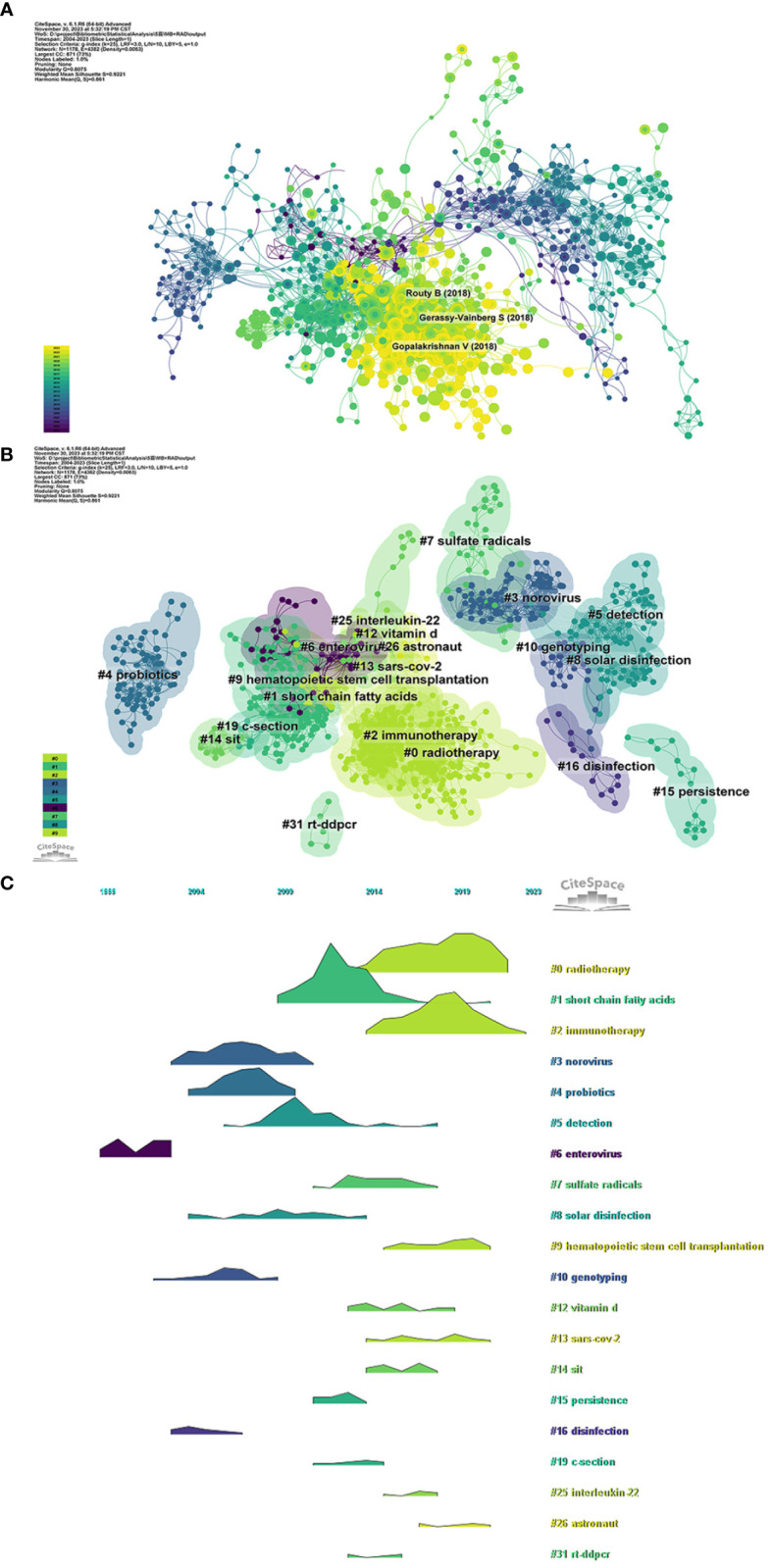
**(A)** Network map of co-cited references obtained with Cite-Space. The nodes represent co-cited references. The lines between the nodes represent cited relationships. The larger the node area, the larger the number of co-citations. The colors from purple to yellow correspond to the annual time periods of publications from 2004 to 2023. **(B)** Cluster analysis of co-cited references obtained with Cite-Space. The color of the node corresponds to various clusters. **(C)** The volcano plot and time-series clustering analysis of co-cited references obtained with Cite-Space.

We examined the time period from 2004 to 2023 and segmented it into one-year intervals for analyzing the dynamic changes in the co-citation network (**Figure 5A**). This network consists of 1178 nodes (representing authors of literature) and 4382 links (representing citation relationships among these nodes). **Table 6** presents the top ten most frequently co-cited references. These investigations highlight the potential role of the gut microbiome in modulating host immune responses, enhancing the effectiveness of cancer treatments, and protecting against RI.

**Table 6:** Top ten co-cited references in research on gut microbiota and radiotherapy.

Rank	Title	Journal IF (2021)	Author(s)	Total citations
1	Gut microbiome influences efficacy of PD-1-based immunotherapy against epithelial tumors	Science (IF=56.9)	Routy B	99
2	Radiation induces proinflammatory dysbiosis: transmission of inflammatory susceptibility by host cytokine induction	Gut (IF=24.5)	Gerassy-Vainberg S	89
3	Gut microbiome modulates response to anti-PD-1 immunotherapy in melanoma patients	Science (IF=56.9)	Gopalakrishnan V	83
4	Fecal microbiota transplantation protects against radiation-induced toxicity	EMBO Mol Med (IF=11.1)	Cui M	64
5	The commensal microbiome is associated with anti-PD-1 efficacy in metastatic melanoma patients	Science (IF=56.9)	Matson V	61
6	Microbiota- and Radiotherapy-Induced Gastrointestinal Side-Effects (MARS) Study: A Large Pilot Study of the Microbiome in Acute and Late-Radiation Enteropathy	Clin Cancer Res (IF=11.5)	Ferreira MR	56
7	Multi-omics analyses of radiation survivors identify radioprotective microbes and metabolites	Science (IF=56.9)	Guo H	52
8	Gut microbial dysbiosis is associated with development and progression of radiation enteritis during pelvic radiotherapy	J Cell Mol Med (IF=5.3)	Wang ZQ	50
9	Commensal Bifidobacterium promotes antitumor immunity and facilitates anti-PD-L1 efficacy	Science (IF=56.9)	Sivan A	42
10	Anticancer immunotherapy by CTLA-4 blockade relies on the gut microbiota	Science (IF=56.9)	Vétizou M	37

Co-citation reference clustering and temporal clustering analyses were performed to identify literature clusters with similar topics, methods, or viewpoints within the research field (**Figures 5B, C**). These findings reveal the changing trends of clusters during specific time periods. Our findings indicate that early research hotspots included enterovirus (cluster 6), norovirus (cluster 3), probiotics (cluster 4), genotyping (cluster 10), and disinfection (cluster 16). Mid-term focal points were short-chain fatty acids (cluster 1), detection (cluster 5), sulfate radicals (cluster 7), solar disinfection (cluster 8), vitamin D (cluster 12), sit (cluster 14), persistence (cluster 15), c-section (cluster 19), interleukin-22 (cluster 25), and rt-ddpcr (cluster 31). Current trending topics and areas of interest in this field are radiotherapy (cluster 0), immunotherapy (cluster 2), hematopoietic stem cell transplantation (cluster 9), SARS-CoV-2 (cluster 13), and astronaut health (cluster 26).

### 3.6 Analysis of keywords

**Figure 6:**
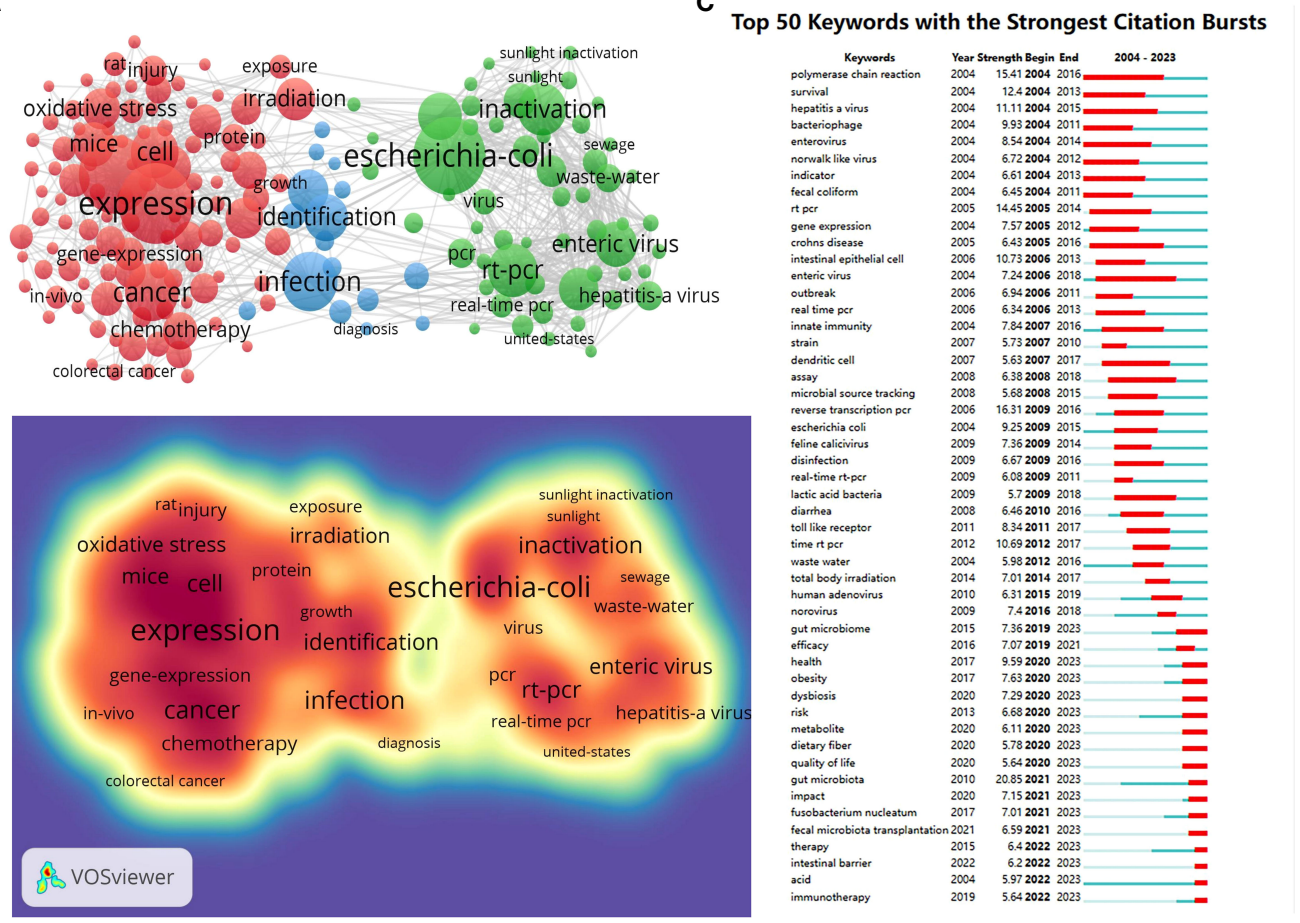
**(A)** A Network map of high-frequency keywords obtained with VOS Viewer. Colors represent clusters automatically calculated by VOSviewer. Each node represents a different keyword. The size of points means the frequency of keywords. **(B)** Density visualization map of keywords obtained with obtained with VOS Viewer, with the keywords in red color area appear more frequently. **(C)** Top 50 keywords with the most robust citation burst obtained with CiteSpace. The blue line indicates the timeline, and the bars in red stands for a burst period including the beginning year, the end year, and the burst duration of the keywords.

Keyword analysis was performed to determine the hotspots in gut microbiota and radiotherapy. According to the co-occurrence of keywords in VOSviewer, the most popular keywords were *expression* (160 occurrences), followed by *Escherichia coli* (151), *inflammation* (135), and *probiotics* (101) as shown in **Table 7** and **Figures 6A, B**. After removing non-informative keywords, we constructed a network comprising 169 keywords that appeared at least 27 times, resulting in three distinct clusters. The first group (red) included 98 keywords such as *expression*, *inflammation*, *probiotics*, *therapy*, *chemotherapy*, *bacterial translocation*, *metabolism*, *health*, *immunity*, *colorectal cancer*, and *efficacy*. The second group (green) contained 53 keywords, including *Escherichia coli*, *rt-pcr*, *norovirus*, *Escherichia coli*, *temperature*, *enteric viruses*, and *murine norovirus*. The third group (blue) comprised 18 keywords, including *infection*, *identification*, *diversity*, *resistance*, *prevalence*, *epidemiology*, *bacteria*, and *diagnosis*.

**Table 7:** Top ten high-frequency keywords in research on gut microbiota and radiotherapy.

Rank	Keyword	Counts	Rank	Keyword	Counts
1	Expression	217	10	Survival	120
2	*Escherichia-coli*	210	12	Enteric Viruses	115
3	Inflammation	209	13	Mice	112
4	Probiotics	184	14	Diversity	109
5	Cancer	151	15	Irradiation	107
6	RT-PCR	138	16	Oxidative Stress	103
7	Inactivation	134	17	*Norovirus*	97
8	Infection	129	18	Disease	96
9	Cells	125	19	Health	93
10	Identification	120	20	Chemotherapy	92

Keyword burst patterns were detected to reveal active contents in the field of gut microbiota and radiotherapy over the past two decades. Among 155 keywords with the strongest citation bursts in this field, the top 50 keywords were shown in **Figure 6C**. We pay special attention to the keywords with a burst period until 2023 among all the burstness keywords because they may represent the current research hotspots in the field and potential directions for future research, such as *gut microbiome*, *health*, *obesity*, *dysbiosis*, *risk*, *metabolite*, *dietary fiber*, *quality of life*, *gut microbiota, impact*, *Fusobacterium nucleatum*, *fecal microbiota transplantation*, *therapy*, *intestinal barrier, acid*, and *immunotherapy.*

## 4 Discussion

The relationship between gut microbiota and its corresponding interventions in radiotherapy is becoming a research focus of scholars. This is the first bibliometric analysis to evaluate and visualize research on gut microbiota and radiotherapy. A total of 2821 publications originating from the WoSCC database were analyzed. We offer a detailed analysis of global trends and hotspots in gut microbiota and radiotherapy research over the past two decades, providing crucial insights into the historical development and research frontiers of this field.

### 4.1 Growth of research interest and general trends in the research field

The number and trend of publications in a certain field can serve as an indicator of its developmental phases. From the annual number of publications, 2004-2010 belonged to the initial stage of research on gut microbiota and radiotherapy, 2010-2017 was in a steady development stage, and 2017-2023 was in a stage of rapid development. It is noteworthy that the year 2010 saw a pivotal turning point with the establishment of a comprehensive catalog of human gut microbial genes through metagenomic sequencing, thereby inaugurating a new trajectory in the field of gut microbiota research. This uptrend suggests an expanding acknowledgment of the microbiome’s role in modulating radiotherapy effects.

The gradual increase in the annual number of publications also reflects the evolving understanding and emerging curiosity in this field. This interest is further reflected in the notable increase in collaborative efforts and the expansion of research across various global institutions. The research landscape has been shaped by contributions from across the globe, with the USA and China being predominant contributors. Such high levels of research output from these two countries can be partly attributed to the high attention and financial support of the government and the community on the gut microbiota program research. the USA initiated the Human Microbiome Project in 2007, followed by the National Microbiome Initiative in 2016. Similarly, China established the Chinese Academy of Sciences Microbiome Program in 2017. Notably, while China exhibited a substantial volume of publications in the field, it is evident that the average citation counts fell significantly short in comparison to other nations, and there was a notable absence of highly cited papers. This observation suggests the imperative for China to continue enhancing the caliber of its scholarly articles.

### 4.2 Current hotspots and field development predictions

Predictions for field development, as extrapolated from the trends in our dataset, indicate a continued interest in investigating how alterations in the gut microbiota due to radiotherapy can lead to various systemic effects, including gastrointestinal, cognitive, and cardiopulmonary dysfunctions, and how to protect against radiation-induced toxicity via gut microbiota, as indicated by the substantial research output from multiple institutions (Cui et al., 2017; Reis Ferreira et al., 2019; Wang et al., 2019; Guo et al., 2020; Ling et al., 2020; Davar et al., 2021; Wang et al., 2023a).

Furthermore, the emergence of certain keywords in our analysis, such as *short-chain fatty acids*, *inflammation*, and *probiotics*, points towards a growing exploration of therapeutic interventions targeting the gut microbiota (Gerassy-Vainberg et al., 2018; Guo et al., 2020; Wang et al., 2021). Such interventions are anticipated to gain more traction in future studies, particularly in mitigating radiation-induced toxicities and enhancing the overall effectiveness of radiotherapy. However, our findings did not indicate a significant relationship between the use of traditional medicine and the main focus of our study. While curcumin has been used to protect gut microbiota, these studies did not emerge as a central theme in the literature we analyzed.

The current hotspots and future trends in this research field, as derived from our bibliometric analysis, suggesting a multi-disciplinary approach that integrates microbiology, oncology, and therapeutics. This approach is pivotal for developing innovative strategies to leverage the gut microbiota in enhancing radiotherapy outcomes and managing radiation-induced complications.

### 4.3 Limitations

While our study provides comprehensive insights, there are inherent limitations. The reliance on bibliometric data from the WoSCC database may omit relevant studies published in other databases or journals not indexed in WoSCC. Furthermore, the analysis is constrained to articles published in English, potentially overlooking significant contributions in other languages. The interpretation of trends and hotspots is subject to the limitations of the bibliometric method, which primarily focuses on quantitative rather than qualitative assessment of the research.

## 5 Conclusions

The intersection of gut microbiota and radiotherapy represents a rapidly evolving research domain, with a clear surge in interest and publications in recent years. This study’s bibliometric analysis has illuminated the growth trends, current hotspots, and potential future directions in this field. The increasing focus on the gut microbiome’s role in radiotherapy underscores its potential as a critical factor in cancer treatment strategies. As the field continues to evolve, it is imperative to overcome current limitations by incorporating diverse research perspectives and methodologies. The insights from this study provide a foundation for future research, encouraging continued exploration and innovation in understanding the complex relationship between gut microbiota and radiotherapy.

## Data Availability

The raw data supporting the conclusions of this article will be made available by the authors, without undue reservation.
