# Supplementary material for: Global research trends on the links between gut microbiota and radiotherapy: a bibliometric analysis (2004-2023)
Source: Front Cell Infect Microbiol. 2024 Sep 4;14:1414196. doi: 10.3389/fcimb.2024.1414196 (PMC11409093; doi:10.3389/fcimb.2024.1414196)
Supplement: Supplementary file 1 [file Datasheet1.pdf]

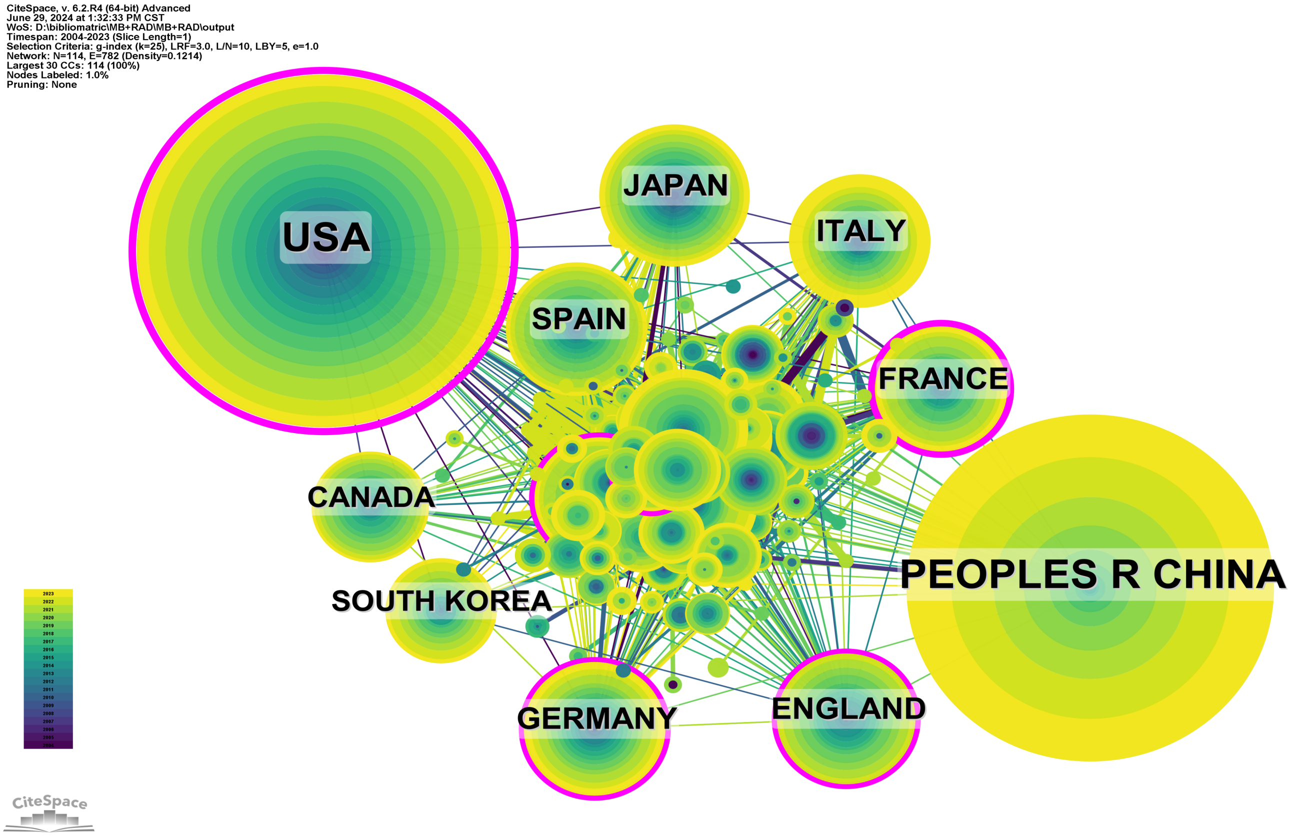
CiteSpace, v. 6.2.R4 (64-bit) Advanced
June 29, 2024 at 1:32:33 PM CST
WoS: D:\bibliomatric\MB+RAD\MB+RAD\output
Timespan: 2004-2023 (Slice Length=1)
Selection Criteria: g-index (k=25), LRF=3.0, L/N=10, LBY=5, e=1.0
Network: N=114, E=782 (Density=0.1214)
Largest 30 CCs: 114 (100%)
Nodes Labeled: 1.0%
Pruning: None
USA
JAPAN
ITALY
SPAIN
FRANCE
CANADA
PEOPLES R CHINA
SOUTH KOREA
GERMANY
ENGLAND
CiteSpace

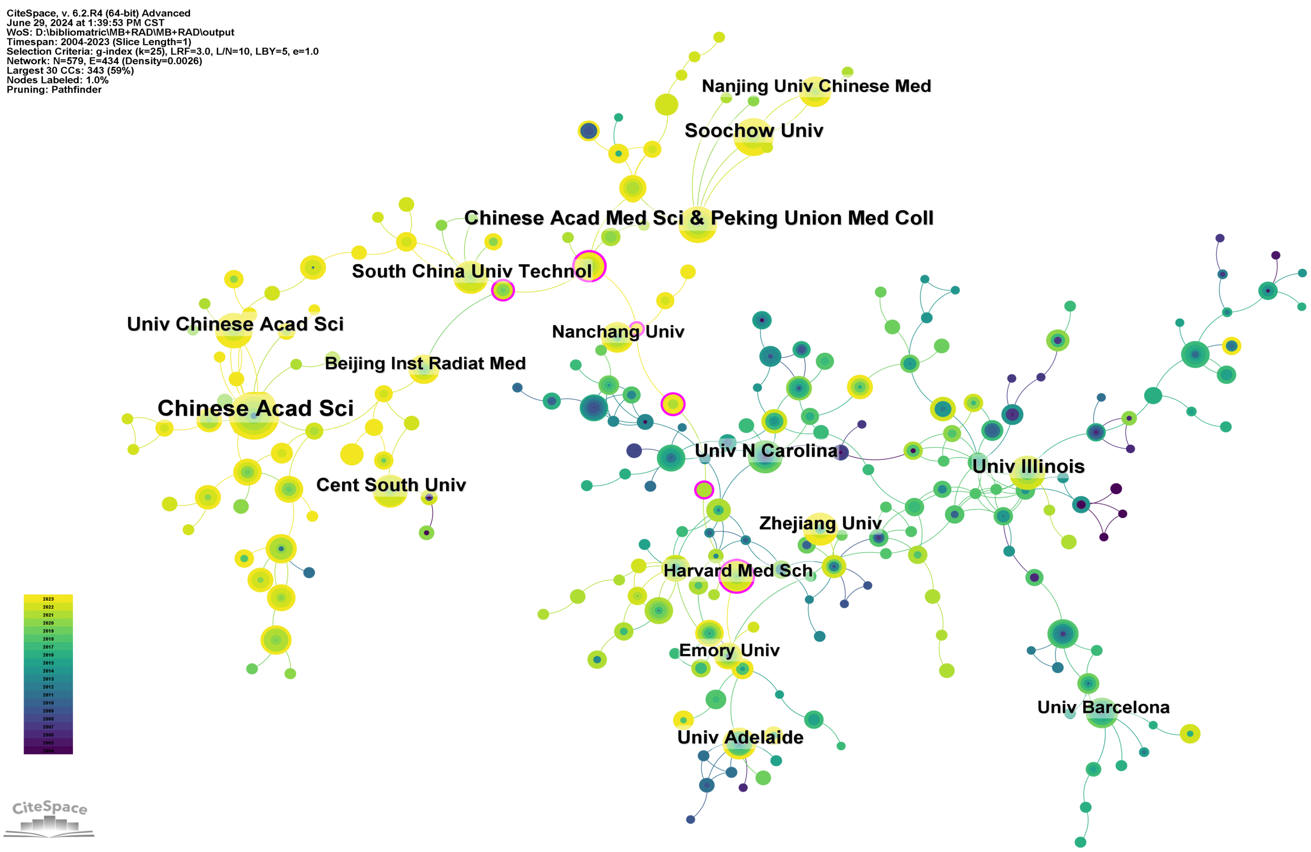
CiteSpace, v. 6.2.R4 (64-bit) Advanced
June 29, 2024 at 1:39:53 PM CST
WoS: D:\bibliomatric\MB+RAD\MB+RAD\output
Timespan: 2004-2023 (Slice Length=1)
Selection Criteria: g-index (k=25), LRF=3.0, L/N=10, LBY=5, e=1.0
Network: N=579, E=434 (Density=0.0026)
Largest 30 CCs: 343 (59%)
Nodes Labeled: 1.0%
Pruning: Pathfinder
Nanjing Univ Chinese Med
Soochow Univ
Chinese Acad Med Sci & Peking Union Med Coll
South China Univ Technol
Univ Chinese Acad Sci
Nanchang Univ
Beijing Inst Radiat Med
Chinese Acad Sci
Univ N Carolina
Univ Illinois
Cent South Univ
Zhejiang Univ
Harvard Med Sch
Emory Univ
Univ Adelaide
Univ Barcelona
CiteSpace

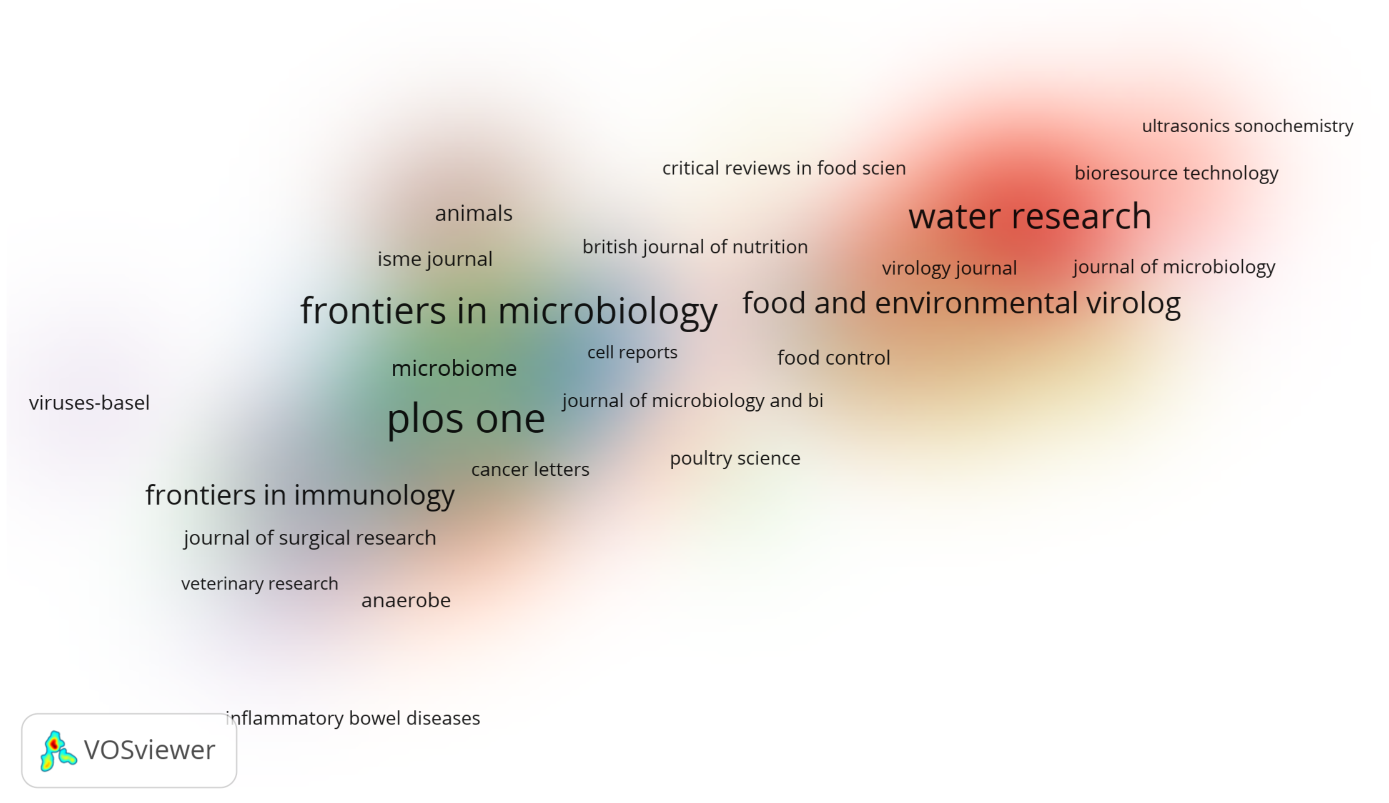
ultrasonics sonochemistry
critical reviews in food scien
bioresource technology
animals
water research
british journal of nutrition
isme journal
virology journal
journal of microbiology
frontiers in microbiology
food and environmental virolog
cell reports
food control
microbiome
viruses-basel
journal of microbiology and bi
plos one
cancer letters
poultry science
frontiers in immunology
journal of surgical research
veterinary research
anaerobe
inflammatory bowel diseases
VOSviewer

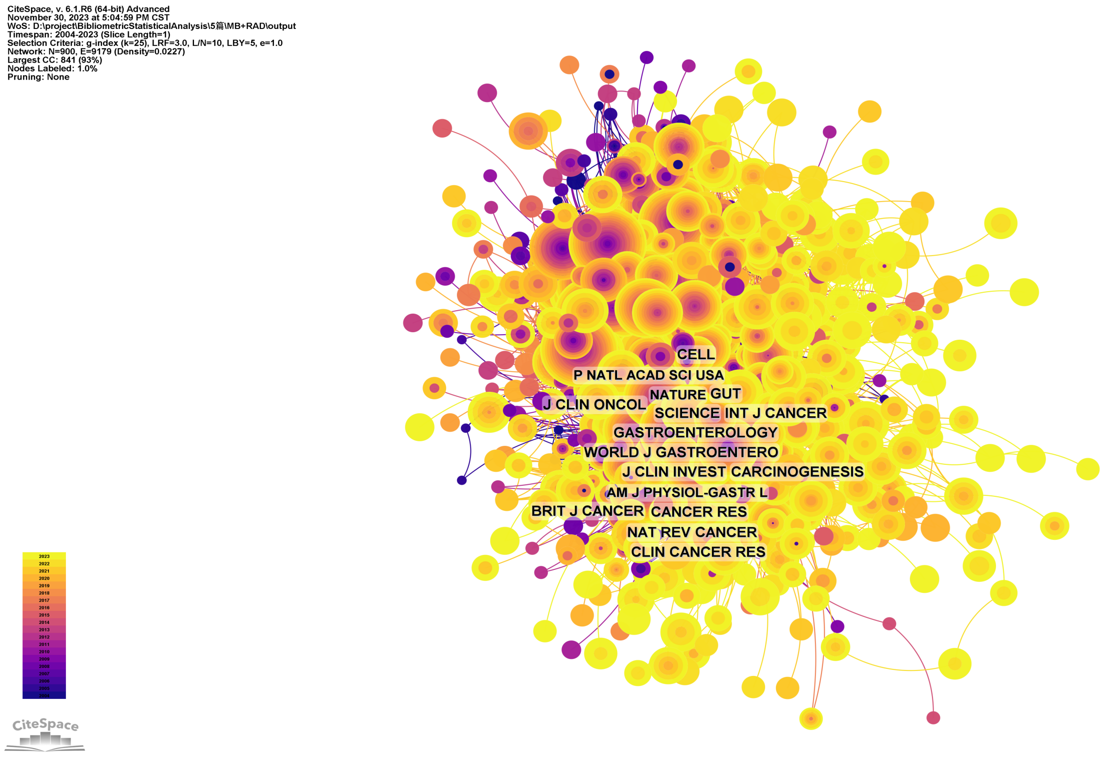

CiteSpace, v. 6.1.R6 (64-bit) Advanced
November 30, 2023 at 5:04:59 PM CST
WoS: D:\project\BibliometricStatisticalAnalysis\5篇\MB+RAD\output
Timespan: 2004-2023 (Slice Length=1)
Selection Criteria: g-index (k=25), LRF=3.0, L/N=10, LBY=5, e=1.0
Network: N=900, E=9179 (Density=0.0227)
Largest CC: 841 (93%)
Nodes Labeled: 1.0%
Pruning: None
CELL
P NATL ACAD SCI USA
NATURE
GUT
J CLIN ONCOL
SCIENCE
INT J CANCER
GASTROENTEROLOGY
WORLD J GASTROENTERO
J CLIN INVEST
CARCINOGENESIS
AM J PHYSIOL-GASTR L
BRIT J CANCER
CANCER RES
NAT REV CANCER
CLIN CANCER RES
CiteSpace

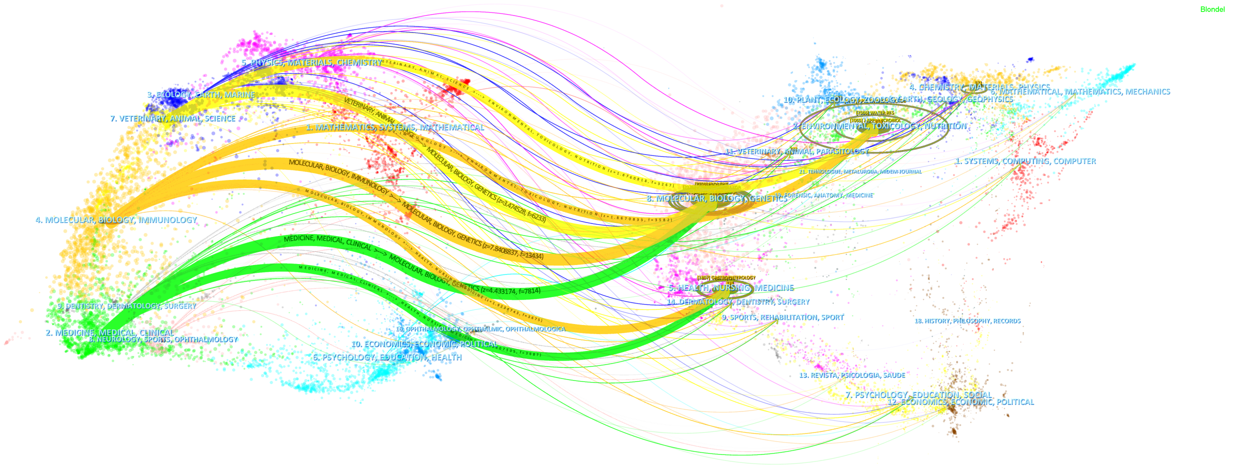

Blondel
5. PHYSICS, MATERIALS, CHEMISTRY
3. ECOLOGY, EARTH, MARINE
7. VETERINARY, ANIMAL, SCIENCE
1. MATHEMATICS, SYSTEMS, MATHEMATICAL
4. MOLECULAR, BIOLOGY, IMMUNOLOGY
9. DENTISTRY, DERMATOLOGY, SURGERY
2. MEDICINE, MEDICAL, CLINICAL
8. NEUROLOGY, SPORTS, OPHTHALMOLOGY
10. ECONOMICS, ECONOMIC, POLITICAL
6. PSYCHOLOGY, EDUCATION, HEALTH
MOLECULAR, BIOLOGY, IMMUNOLOGY ---> MOLECULAR, BIOLOGY, GENETICS (z=7.8408837, f=13434)
MEDICINE, MEDICAL, CLINICAL ---> MOLECULAR, BIOLOGY, GENETICS (z=4.433174, f=7814)
10. PLANT, ECOLOGY, ZOOLOGY
4. CHEMISTRY, MATERIALS, PHYSICS
6. EARTH, GEOLOGY, GEOPHYSICS
1. MATHEMATICAL, MATHEMATICS, MECHANICS
2. ENVIRONMENTAL, TOXICOLOGY, NUTRITION
11. VETERINARY, ANIMAL, PARASITOLOGY
3. SYSTEMS, COMPUTING, COMPUTER
8. MOLECULAR, BIOLOGY, GENETICS
5. HEALTH, NURSING, MEDICINE
14. DERMATOLOGY, DENTISTRY, SURGERY
9. SPORTS, REHABILITATION, SPORT
18. HISTORY, PHILOSOPHY, RECORDS
13. REVISTA, PSICOLOGIA, SAUDE
7. PSYCHOLOGY, EDUCATION, SOCIAL
12. ECONOMICS, ECONOMIC, POLITICAL

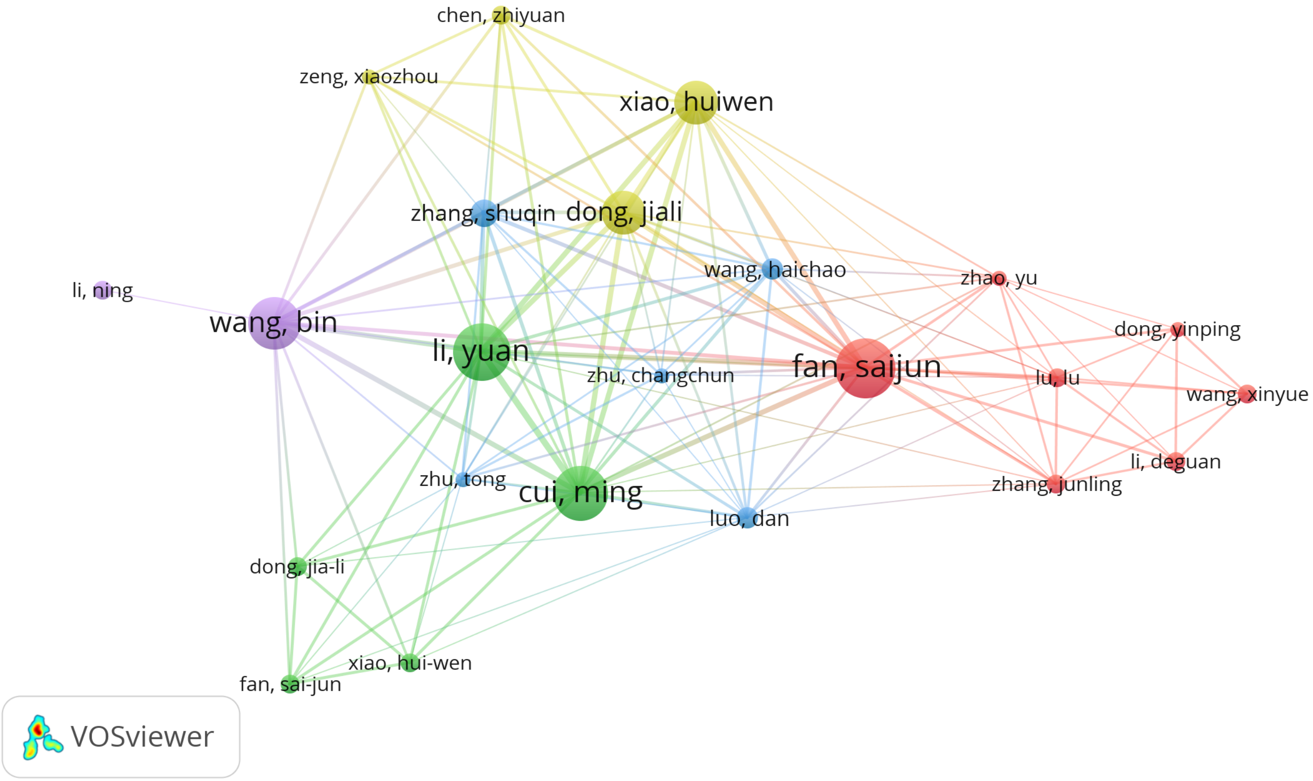
chen, zhiyuan
zeng, xiaozhou
xiao, huiwen
zhang, shuqin
dong, jiali
wang, haichao
zhao, yu
li, ning
wang, bin
li, yuan
dong, yinping
zhu, changchun
fan, saijun
lu, lu
wang, xinyue
li, deguan
zhu, tong
cui, ming
zhang, junling
luo, dan
dong, jia-li
xiao, hui-wen
fan, sai-jun
VOSviewer

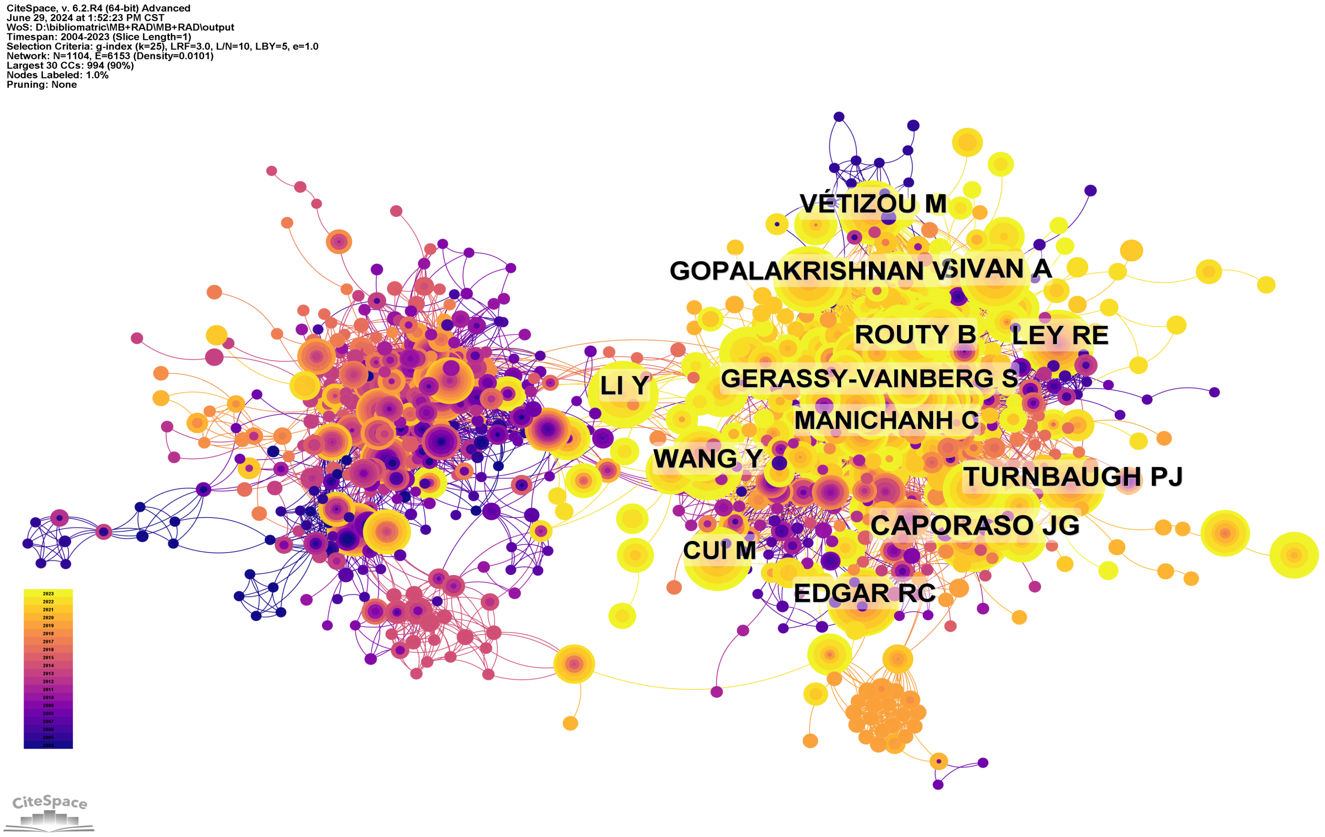
CiteSpace, v. 6.2.R4 (64-bit) Advanced
June 29, 2024 at 1:52:23 PM CST
WoS: D:\bibliomatric\MB+RAD\MB+RAD\output
Timespan: 2004-2023 (Slice Length=1)
Selection Criteria: g-index (k=25), LRF=3.0, L/N=10, LBY=5, e=1.0
Network: N=1104, E=6153 (Density=0.0101)
Largest 30 CCs: 994 (90%)
Nodes Labeled: 1.0%
Pruning: None
VÉTIZOU M
GOPALAKRISHNAN V
SIVAN A
ROUTY B
LEY RE
LI Y
GERASSY-VAINBERG S
MANICHANH C
WANG Y
TURNBAUGH PJ
CAPORASO JG
CUI M
EDGAR RC
CiteSpace
