# Supplementary material for: Global research trends on the links between gut microbiota and radiotherapy: a bibliometric analysis (2004-2023)
Source: Front Cell Infect Microbiol. 2024 Sep 4;14:1414196. doi: 10.3389/fcimb.2024.1414196 (PMC11409093; doi:10.3389/fcimb.2024.1414196)
Supplement: Supplementary file 2 [file Datasheet2.pdf]

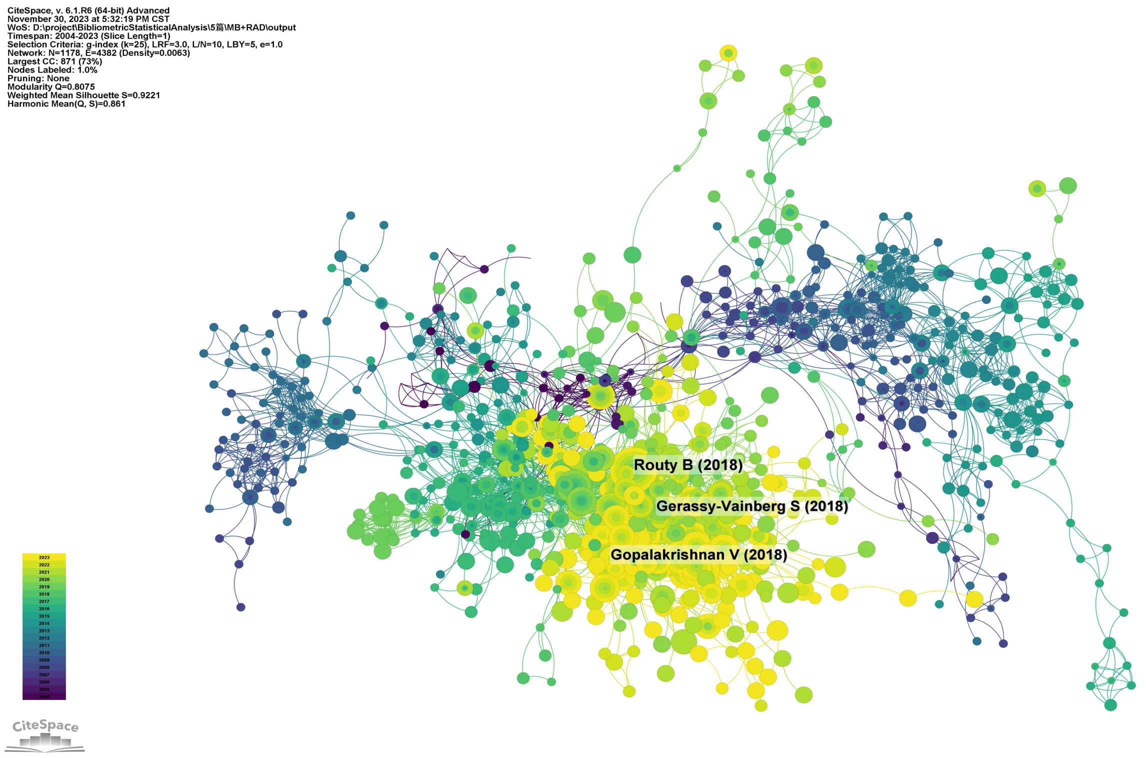
CiteSpace, v. 6.1.R6 (64-bit) Advanced
November 30, 2023 at 5:32:19 PM CST
WoS: D:\project\BibliometricStatisticalAnalysis\5篇\MB+RAD\output
Timespan: 2004-2023 (Slice Length=1)
Selection Criteria: g-index (k=25), LRF=3.0, L/N=10, LBY=5, e=1.0
Network: N=1178, E=4382 (Density=0.0063)
Largest CC: 871 (73%)
Nodes Labeled: 1.0%
Pruning: None
Modularity Q=0.8075
Weighted Mean Silhouette S=0.9221
Harmonic Mean(Q, S)=0.861
Routy B (2018)
Gerassy-Vainberg S (2018)
Gopalakrishnan V (2018)
CiteSpace

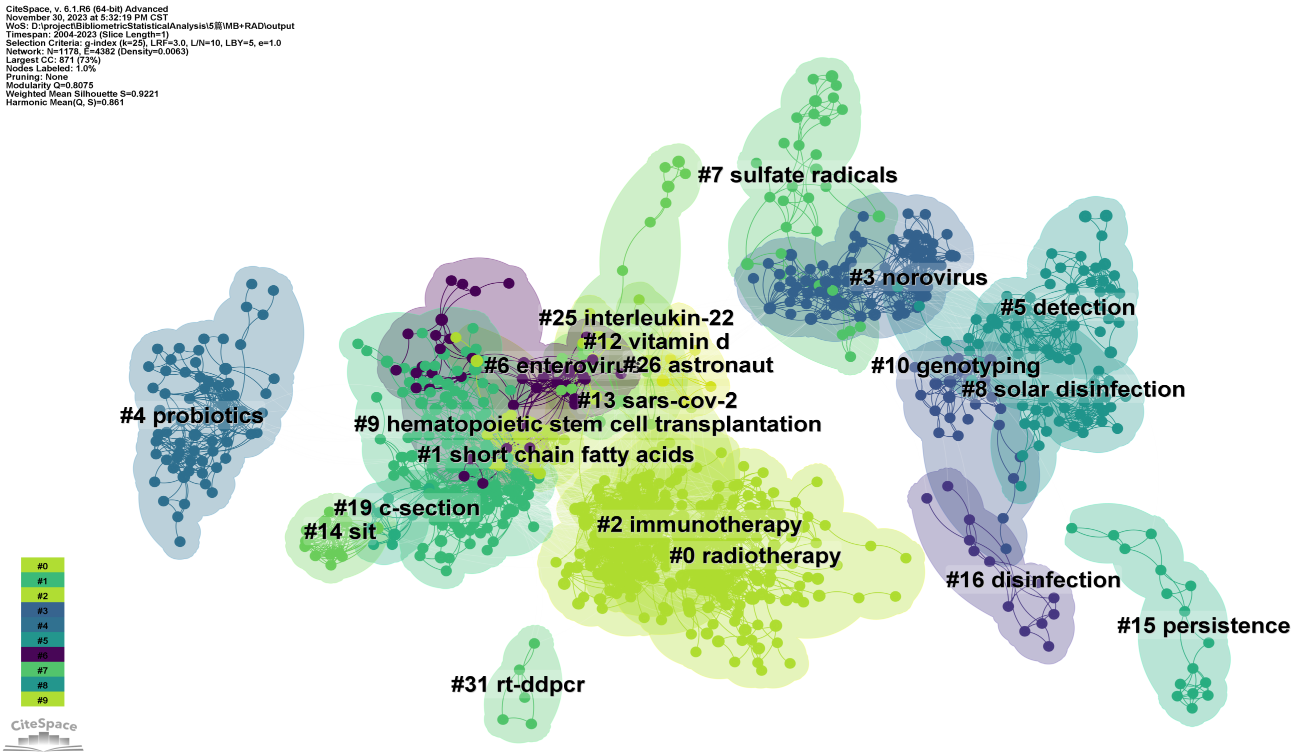
CiteSpace, v. 6.1.R6 (64-bit) Advanced
November 30, 2023 at 5:32:19 PM CST
WoS: D:\project\BibliometricStatisticalAnalysis\5篇\MB+RAD\output
Timespan: 2004-2023 (Slice Length=1)
Selection Criteria: g-index (k=25), LRF=3.0, L/N=10, LBY=5, e=1.0
Network: N=1178, E=4382 (Density=0.0063)
Largest CC: 871 (73%)
Nodes Labeled: 1.0%
Pruning: None
Modularity Q=0.8075
Weighted Mean Silhouette S=0.9221
Harmonic Mean(Q, S)=0.861
#7 sulfate radicals
#3 norovirus
#5 detection
#25 interleukin-22
#12 vitamin d
#6 enterovirus
#26 astronaut
#10 genotyping
#8 solar disinfection
#4 probiotics
#13 sars-cov-2
#9 hematopoietic stem cell transplantation
#1 short chain fatty acids
#19 c-section
#14 sit
#2 immunotherapy
#0 radiotherapy
#16 disinfection
#15 persistence
#31 rt-ddpcr
#0
#1
#2
#3
#4
#5
#6
#7
#8
#9
CiteSpace

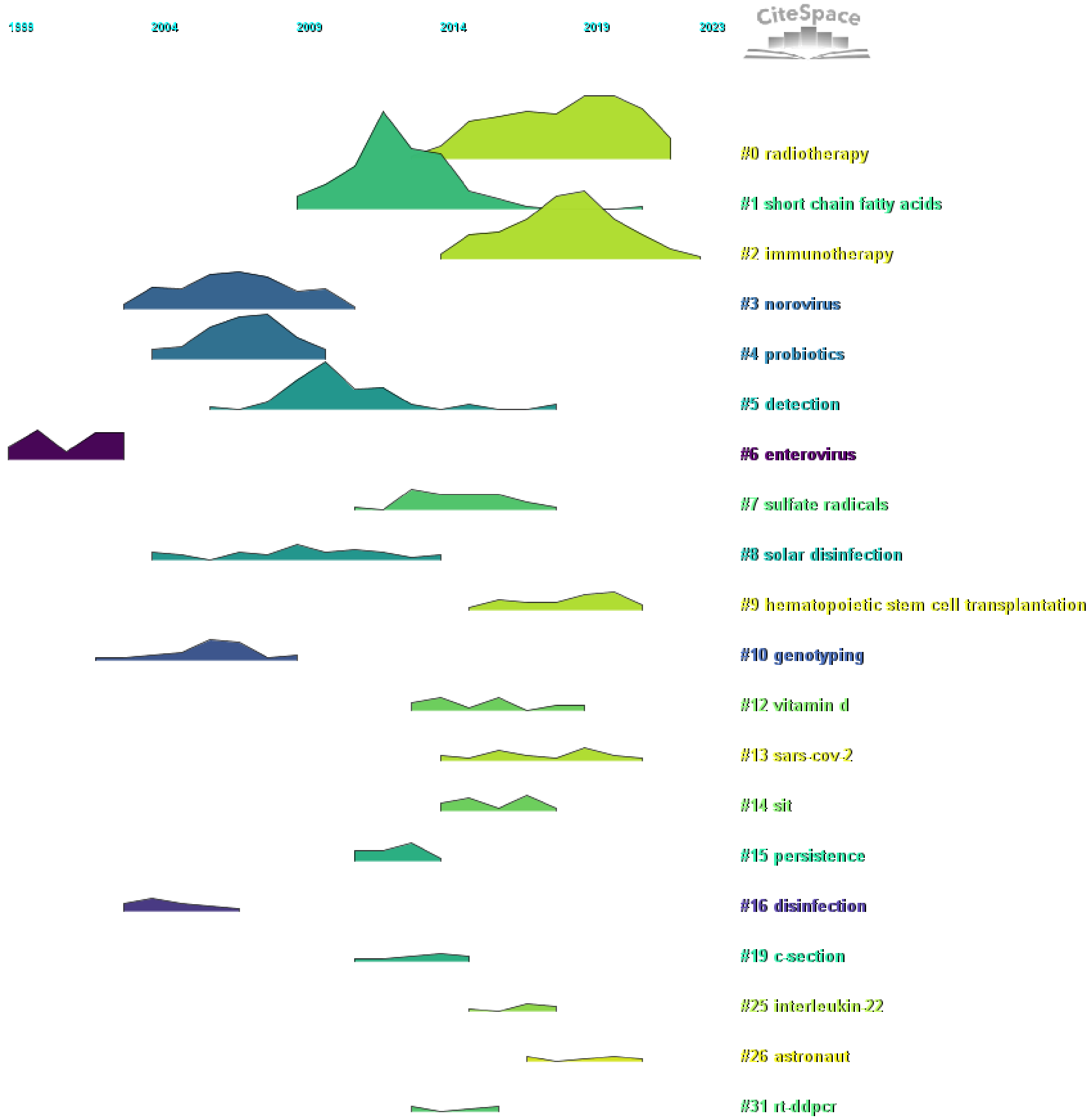
1999
2004
2009
2014
2019
2023
CiteSpace
#0 radiotherapy
#1 short chain fatty acids
#2 immunotherapy
#3 norovirus
#4 probiotics
#5 detection
#6 enterovirus
#7 sulfate radicals
#8 solar disinfection
#9 hematopoietic stem cell transplantation
#10 genotyping
#12 vitamin d
#13 sars-cov-2
#14 sit
#15 persistence
#16 disinfection
#19 c-section
#25 interleukin-22
#26 astronaut
#31 rt-ddpcr

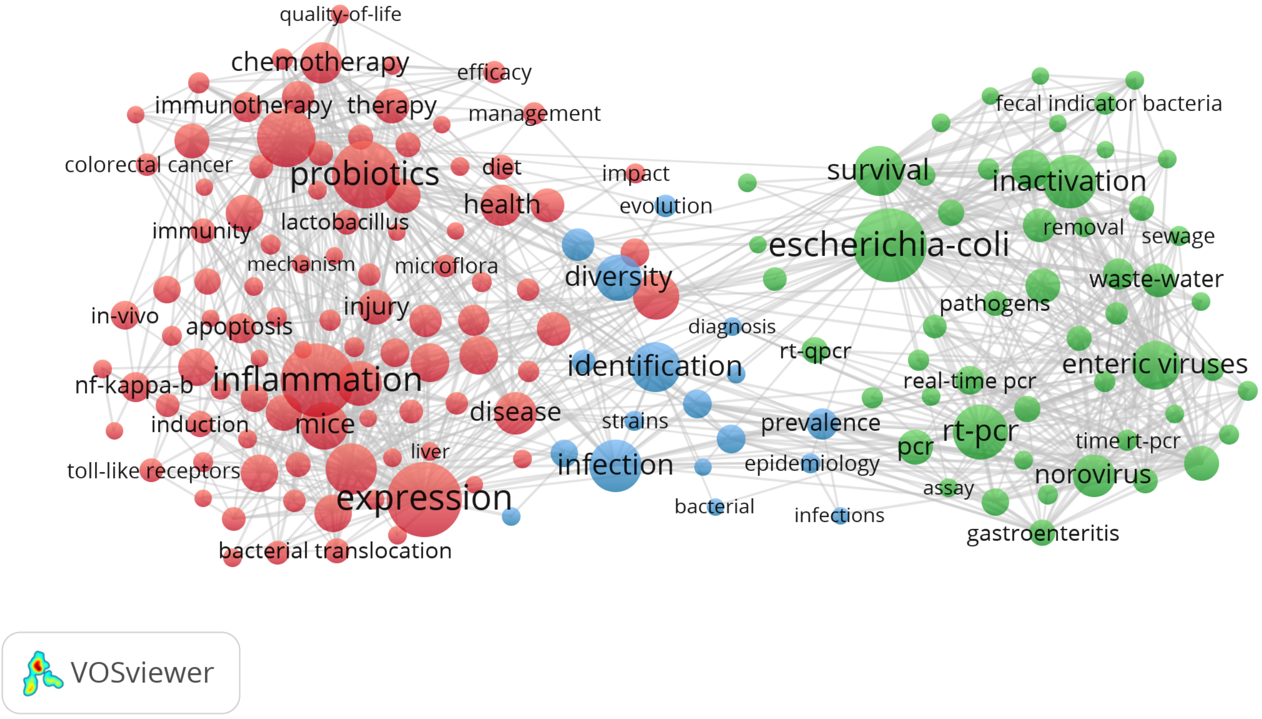
quality-of-life
chemotherapy
efficacy
immunotherapy
therapy
management
colorectal cancer
probiotics
diet
impact
health
evolution
immunity
lactobacillus
mechanism
microflora
diversity
in-vivo
injury
apoptosis
diagnosis
nf-kappa-b
inflammation
identification
induction
mice
disease
strains
toll-like receptors
liver
infection
expression
bacterial
bacterial translocation
survival
inactivation
fecal indicator bacteria
escherichia-coli
removal
sewage
waste-water
pathogens
rt-qpcr
real-time pcr
enteric viruses
prevalence
epidemiology
pcr
rt-pcr
time rt-pcr
norovirus
assay
infections
gastroenteritis
VOSviewer

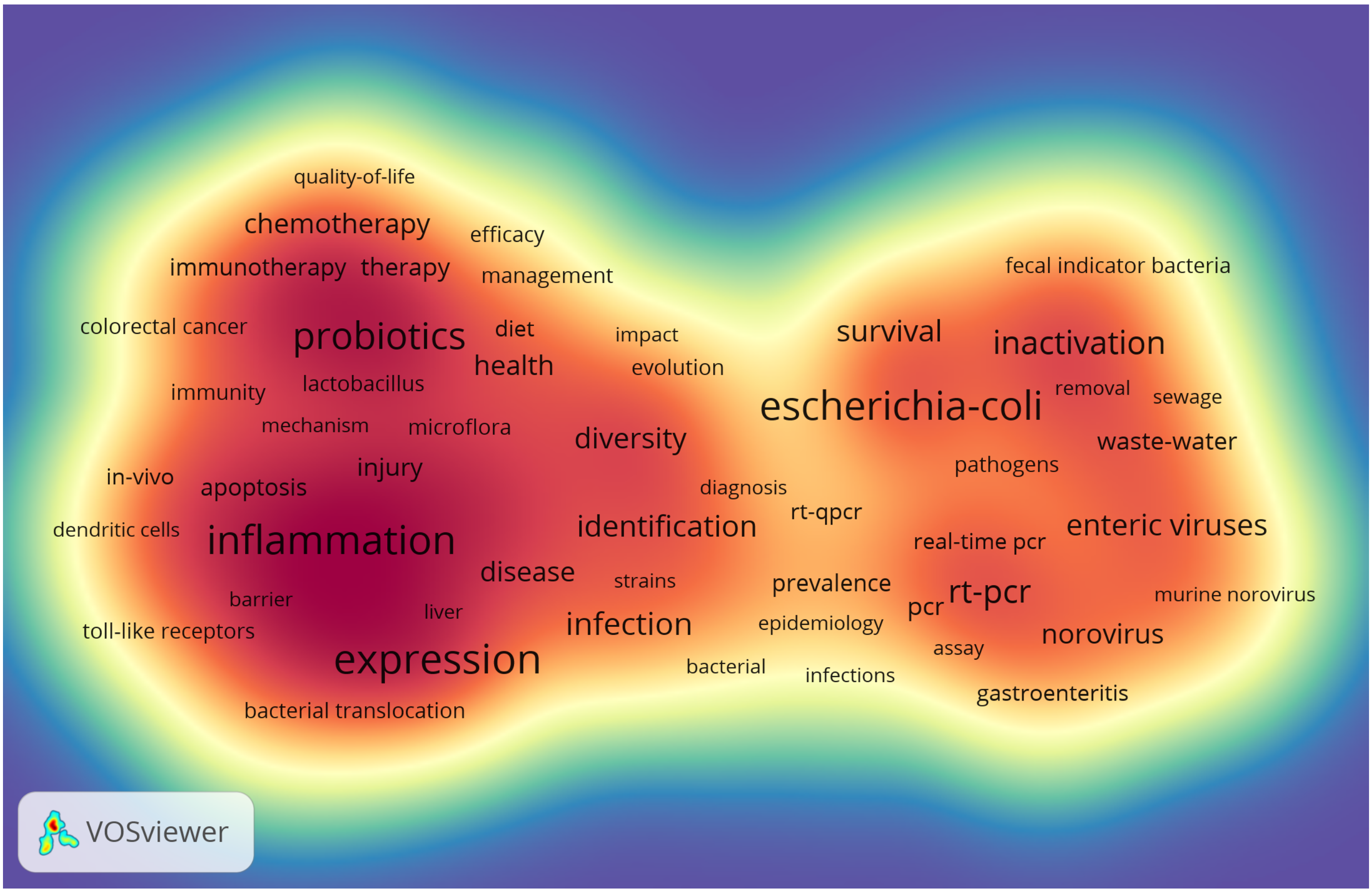

quality-of-life
chemotherapy
efficacy
immunotherapy
therapy
management
fecal indicator bacteria
colorectal cancer
probiotics
diet
impact
survival
inactivation
health
evolution
immunity
lactobacillus
escherichia-coli
removal
sewage
mechanism
microflora
diversity
waste-water
injury
pathogens
in-vivo
apoptosis
diagnosis
dendritic cells
inflammation
identification
rt-qpcr
real-time pcr
enteric viruses
disease
strains
prevalence
barrier
liver
pcr
rt-pcr
murine norovirus
toll-like receptors
infection
epidemiology
norovirus
expression
assay
bacterial
infections
gastroenteritis
bacterial translocation
VOSviewer

Top 50 Keywords with the Strongest Citation Bursts

Keywords	Year	Strength	Begin	End	2004 - 2023
polymerase chain reaction	2004	15.41	**2004**	2016	
survival	2004	12.4	**2004**	2013	
hepatitis a virus	2004	11.11	**2004**	2015	
bacteriophage	2004	9.93	**2004**	2011	
enterovirus	2004	8.54	**2004**	2014	
norwalk like virus	2004	6.72	**2004**	2012	
indicator	2004	6.61	**2004**	2013	
fecal coliform	2004	6.45	**2004**	2011	
rt pcr	2005	14.45	**2005**	2014	
gene expression	2004	7.57	**2005**	2012	
crohns disease	2005	6.43	**2005**	2016	
intestinal epithelial cell	2006	10.73	**2006**	2013	
enteric virus	2004	7.24	**2006**	2018	
outbreak	2006	6.94	**2006**	2011	
real time pcr	2006	6.34	**2006**	2013	
innate immunity	2004	7.84	**2007**	2016	
strain	2007	5.73	**2007**	2010	
dendritic cell	2007	5.63	**2007**	2017	
assay	2008	6.38	**2008**	2018	
microbial source tracking	2008	5.68	**2008**	2015	
reverse transcription pcr	2006	16.31	**2009**	2016	
escherichia coli	2004	9.25	**2009**	2015	
feline calicivirus	2009	7.36	**2009**	2014	
disinfection	2009	6.67	**2009**	2016	
real-time rt-pcr	2009	6.08	**2009**	2011	
lactic acid bacteria	2009	5.7	**2009**	2018	
diarrhea	2008	6.46	**2010**	2016	
toll like receptor	2011	8.34	**2011**	2017	
time rt pcr	2012	10.69	**2012**	2017	
waste water	2004	5.98	**2012**	2016	
total body irradiation	2014	7.01	**2014**	2017	
human adenovirus	2010	6.31	**2015**	2019	
norovirus	2009	7.4	**2016**	2018	
gut microbiome	2015	7.36	**2019**	2023	
efficacy	2016	7.07	**2019**	2021	
health	2017	9.59	**2020**	2023	
obesity	2017	7.63	**2020**	2023	
dysbiosis	2020	7.29	**2020**	2023	
risk	2013	6.68	**2020**	2023	
metabolite	2020	6.11	**2020**	2023	
dietary fiber	2020	5.78	**2020**	2023	
quality of life	2020	5.64	**2020**	2023	
gut microbiota	2010	20.85	**2021**	2023	
impact	2020	7.15	**2021**	2023	
fusobacterium nucleatum	2017	7.01	**2021**	2023	
fecal microbiota transplantation	2021	6.59	**2021**	2023	
therapy	2015	6.4	**2022**	2023	
intestinal barrier	2022	6.2	**2022**	2023	
acid	2004	5.97	**2022**	2023	
immunotherapy	2019	5.64	**2022**	2023	
