# Supplementary material for: Global research trends on the links between gut microbiota and radiotherapy: a bibliometric analysis (2004-2023)
Source: Front Cell Infect Microbiol. 2024 Sep 4;14:1414196. doi: 10.3389/fcimb.2024.1414196 (PMC11409093; doi:10.3389/fcimb.2024.1414196)
Supplement: Supplementary file 3 [file Table1.docx]

**Search strategy**：

TS = (“Gut Microbio*” OR “Gut MicroflORa” OR “Gut FlORa” OR “Gut Microbial FlORa” OR “Intestinal Microbio*” OR “Intestinal MicroflORa” OR “Intestinal FlORa” OR “Intestinal Microbial FlORa” OR “Gastrointestinal Microbio*” OR “Gastrointestinal MicroflORa” OR “Gastrointestinal FlORa” OR “Gastrointestinal Microbial FlORa” OR “Gastrointestinal Microbial Communit*” OR “Fecal Microbio*” OR “Fecal MicroflORa” OR “Fecal FlORa” OR “Fecal Microbial FlORa” OR “Faecal Microbio*” OR “Faecal MicroflORa” OR “Faecal FlORa” OR “Faecal Microbial FlORa” OR “Gut Bacteri*” OR “Intestinal Bacteri*” OR “Gastrointestinal Bacteri*” OR “Fecal Bacteri*” OR “Faecal Bacteri*” OR “Enteric Bacteri*”) AND TS = (“radiotherapy” OR “RT” OR “radiation therapy” OR “radiation treatment” OR “irradiation” OR “radiation” OR “SABR” OR “stereotactic ablative radiotherapy” OR “SBRT” OR “stereotactic body radiation therapy” OR “SRS” OR “stereotactic radiosurgery” OR “SRT” OR “stereotactic radiotherapy”)
